# Anion Transport Using Core Functionalized Hyperbranched Polymers and Evidence of a Dense Packed Limit Based on Molecular Weight

**DOI:** 10.3390/molecules26226850

**Published:** 2021-11-13

**Authors:** Sozan Najib Abdullah, Georgia Mann, Lance J. Twyman

**Affiliations:** Department of Chemistry, University of Sheffield, Dainton Building, Sheffield S3 7HF, UK; sozan.abdulla@univsul.edu.iq (S.N.A.); manngeorgia1@gmail.com (G.M.)

**Keywords:** dendritic, hyperbranched polymer, anion transport, dense packing, phase transfer

## Abstract

Being able to bind, select, and transport species is central to a number of fields, including medicine, materials, and environmental science. In particular, recognizing a specific species from one phase and transporting it across, or into another phase, has obvious applications in environ-mental science, for example, removal of unwanted or toxic materials from an aqueous or organic phase. In this paper, we describe an approach that uses a functionalized dendritic polymer to bind and transport a small anionic molecule across an organic phase (and between two aqueous phases). The design was based on encapsulation principles borrowed from nature, where anions are bound and transported by proteins that have specific sites within their globular ordered structures. For the work reported here, a globular dendritic polymer functionalized with an isophthalamide-based receptor was used to replace the protein structure and anion-binding site. Along with control experiments, the binding and transport properties of two functionalized HBPs were assessed using a Pressman U tube experiment. Both HBPs demonstrated an enhanced ability to bind and transport anions (when compared to the anion-binding site used in isolation). Furthermore, optimum binding and transport occurred when the smaller of the two HBPs were used. This supports our previous observations regarding the existence of a dense packed limit for HBPs.

## 1. Introduction

Anions are ubiquitous in numerous biological systems, with both positive and negative effects. For example, epilepsy, myotonia, and cystic fibrosis are all examples of diseases that arise from the misregulation of chloride ion flux [1,2]. Anions can also be potentially threatening to the environment; for instance, nitrate anions present in fertilizers run off the land and pollute water supplies, which brings about excessive algae production that can damage aquatic life [3]. Therefore, the successful and specific coordination of anions has both therapeutic and environmental importance. A good example of anions having a positive biological effect is alkaline phosphatase, which possesses an anionic phosphate binding site surrounded by a protein structure [4]. The active site includes an arginine residue, two zinc ions, and a magnesium ion, affording an anion-binding cavity supported by the large macromolecular structure of the protein. The same encapsulation and supramolecular principles utilized by nature to bind, recognize, and transport guest molecules can be replicated using dendritic polymers. For example, we have reported on a number of dendritic polymers that can mimic the globular structure and function of proteins using their terminal groups [5,6] or through functionalization of their interior or core units [7,8]. In this paper, we describe how a series of dendritic polymers functionalized with an anion-binding site can be used to bind and transport water-soluble anions between aqueous and organic phases.

Dendritic polymers are a class of globular macromolecules possessing well-controlled 3D structures [9]. Two specific classes of dendritic polymer exist. The first are a series of perfectly branched molecules that are synthesized via iterative synthetic steps; these systems are known as dendrimers. Although dendrimers are perfect with respect to size, molecular weight, and structure, they are laborious and expensive to synthesize. An alternative class of branched/dendritic polymer, known as hyperbranched polymers (HBPs) [10], can be synthesized in a single synthetic step using monomers with multiple functionality and standard polymer techniques. Although they are much simpler to construct, they have imperfections with respect to their branching and therefore lack the perfect mono dispersed structure of a dendrimer. Nevertheless, HBPs possess a defined globular 3D structure that is very similar to that of a dendrimer. As a result of these synthetic advantages, HBPs were selected as the host molecules for the work described in this paper. In order for them to be fully utilized as mimics of protein based binding systems, it is essential that HBPs can be functionalized with suitable binding/receptor units at their core. It is possible to achieve this with high core-loading, when complementary core and monomer units are polymerized under reversible conditions [11,12]. We have used this approach to successfully synthesize an HBP with an anion-binding core. This HBP was capable of binding a variety of anions, where selectivity was subsequently moderated by the globular polymer structure [13]. In this work, we wanted to investigate whether the same HBP could bind and transport a water-soluble anion across an organic phase via a facilitated diffusion. This can best be determined using a simple U-tube experiment [14,15], as represented in Figure 3 below. In a secondary aim, we also wanted to reinforce our earlier work on the extent and existence of dense packing limits for HBPs (of different molecular weight/size).

## 2. Results and Discussion

When designing our biomimetic system for anion binding and transport, we considered three main components. Firstly, we considered the specific macromolecular scaffold that would support a binding site and therefore provide the binding environment (the internal globular environment/space). Secondly, we needed to identify a specific binding group that could be easily incorporated within the dendritic carrier. Finally, and in combination with selecting a specific binding group, we also needed to think about the target anionic guest. Specifically, the binding site and target anion must be complimentary with respect to their interactions.

**Scheme 1:**
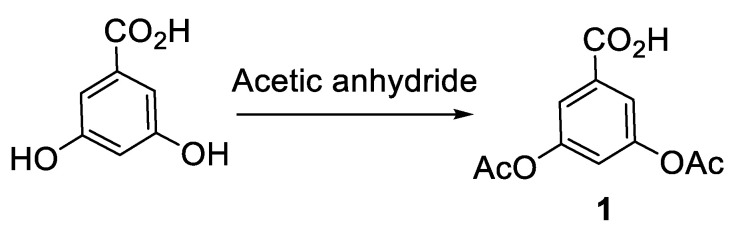
Synthesis of the AB2 monomer 3,5-diacetoxybenzoic acid **1**.

In order to mimic the globular structure of an anion-binding protein, we planned to use a hyperbranched polyarylester based on the AB_2_ repeat unit 3,5-diacetoxybenzoic acid **1 [16]**. 3,5-diacetoxybenzoic acid **1** has two esters and a single carboxylic acid and is commercially available. Alternatively, it can be synthesized easily from 3,5-dihydroxybenzoic acid as shown in Scheme 1. We have used this monomer previously to synthesize core functionalized HBP, including oxygen-binding [7] catalytic [8], and anion-binding cores [13]. The polymerization process involves a reversible trans-esterification. When polymerized on its own, a polydisperse polymer is generated. However, when a suitable core unit is added, the reversible nature of the polymerization provides excellent levels of control with respect to molecular weight, levels of core incorporation, and dispersity [11,12]. Suitable core units that ensure just one core can be incorporated include anything with acetoxy groups (the A part of the AB_2_ monomer). Alternatively, many functional/reactive units can be incorporated using cores with carboxylic acid groups (the B part of the AB_2_ monomer) [17]. Although this second approach can yield a multifunctional HBP, any control with respect to molecular weight and dispersity is lost. As a control was important, we decided to use an acetoxy functionalized core to generate a HBP with a single anion-binding core.

**Figure 1:**
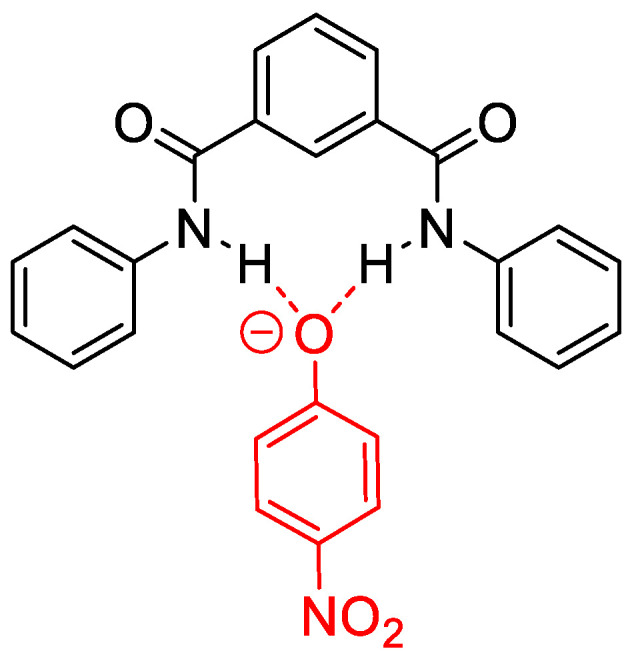
An isophthalamide-based receptor shown binding *p*-nitrophenolate.

When selecting the anion-binding unit, we must also consider the target anion. Although a wide variety of anions could have been selected, we wanted one that simplified analysis, allowing us to easily demonstrate a proof of principle with respect to a biomimetic anion-binding and transport system. Ideally, the anion should be colored, UV-active, and completely soluble in water, whilst possessing properties enabling it to interact with the HBP system. As such, *p*-nitrophenolate was selected. In basic solution, it is bright yellow, water soluble, and possesses a hydrophobic aromatic ring that may contribute to and strengthen binding (through π–π interactions with the aromatic back bone of the HBP). Furthermore, it is known that this anion can bind to isophthalamide based receptors such as **2**, Figure 1 [18].

**Figure 2:**
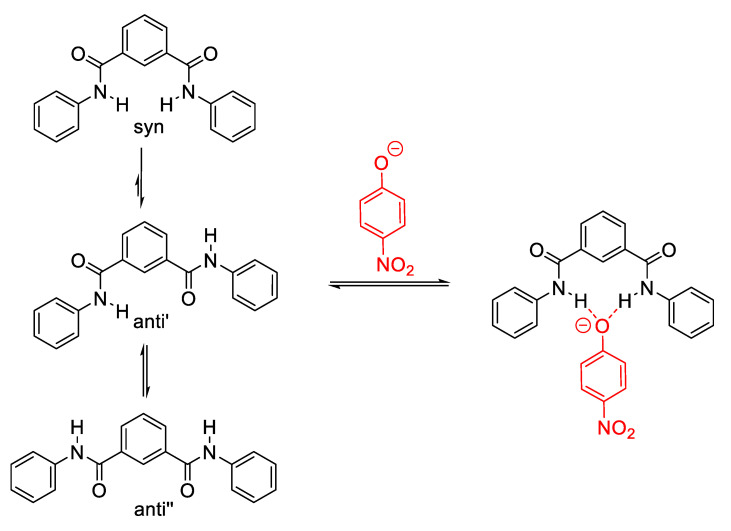
Synthesis of isophthalamide receptor/core unit. Some of the rotational isomers are also shown, including the optimum binding, but least stable, syn configuration (shown binding *p-*nitrophenolate).

**Scheme 2:**
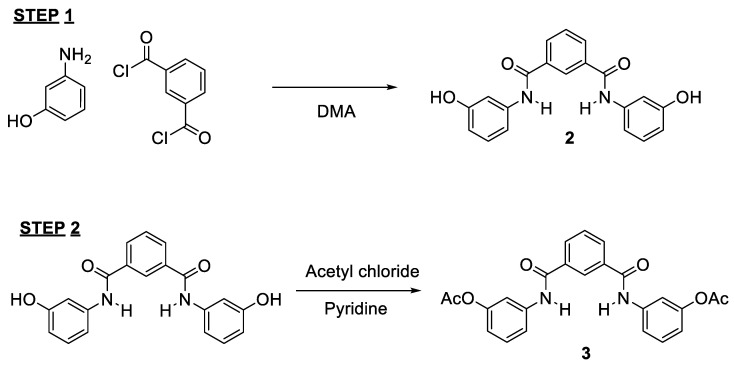
The two-step synthesis of the anion-binding core unit *N,N’-bis*-(3-acetoxy-phenyl)-isophthalamide **3**.

One final consideration concerns the dynamic nature of the isophthalamide, which can exist in a number of rotational isomers, as shown in Figure 2 [19]. Specifically, this receptor must be in the correct rotameric form to bind anions with a high level of affinity. Specifically, isophthalamide needs to be in the syn/syn conformation as shown in Figure 2. As it stands, isophthalamide **2** does not possess the required acetocxy group (for core incorporation within a HBP), and *N*,*N*’-*bis*-(3-acetoxy-phenyl)-isophthalamide **3** was identified as a possible core unit, Scheme 2. This is a symmetrical molecule, which simplifies synthesis and possess 2 acetoxy groups, each of which can initiate polymerization, therefore generating HBP with an anion-binding core at the center of its globular structure.

*N,N’-bis-*(3-acetoxy-phenyl)-isophthalamide **3** was synthesized according to a modified experimental route developed by Malone [20]. 3-aminophenol **4** and *N,N’-*dimethylacetamide **5** were reacted with isophthaloyl chloride at 0 °C over a 6 h period. The intermediate product *N,N’*-*bis*-(3-hydroxy-phenyl)-isophthalamide **6** was obtained in excellent yield after purification. The ^1^H NMR spectrum had singlets at 9.47 and 10.31 ppm, corresponding to the NH and OH protons, respectively. The expected molecular ion peak at 348 was detected via mass spectrometry, and elemental analysis provided good evidence for purity. The final step in the synthesis was acetylation of the hydroxy terminal groups, which was achieved by reacting *N,N’*-*bis*-(3-hydroxy-phenyl)-isophthalamide **6** with acetyl chloride in the presence of pyridine. The reaction was worked up and the crude product purified by recrystallization to give the target compound *N,N’-bis*-(3-acetoxy-phenyl)-isophthalamide **3** in excellent yield. The peak corresponding to the OH protons was no longer visible in the ^1^H NMR and IR spectra. In addition, ^1^H NMR confirmed acetylation had been successful as a new peak at 2.30 ppm, corresponding to the six acetoxy protons, was now visible. Subsequent mass spectrometry analysis showed a molecular ion at 433 and confirmed successful synthesis of the required *N,N’-bis*-(3-acetoxy-phenyl)-isophthalamide **3**.

**Figure 3:**
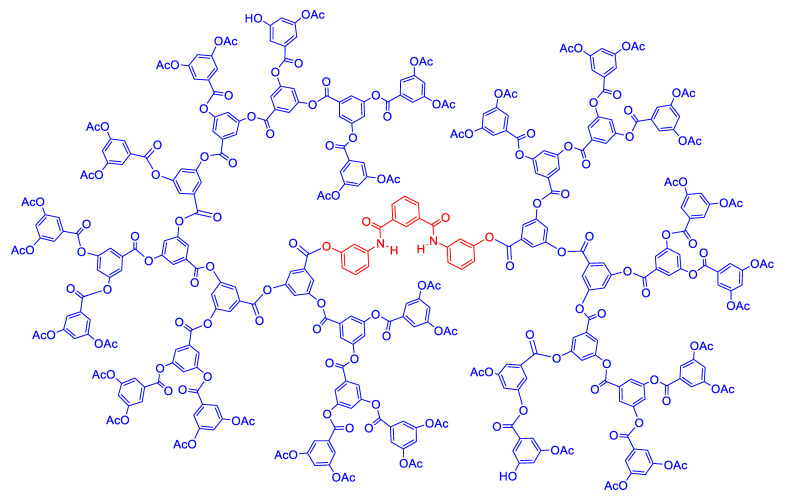
A representative structure of the low molecular weight HBP target (molecular weight between 7000 and 8000). The anion-binding isophthalamide core is shown in its favorable syn conformation.

Having successfully synthesized the core isophthalamide **3**, the next step was to incorporate it into the hyperbranched polymer. In order to examine any effects of steric crowding (dense packing effects) and to obtain an optimized polymer for anion binding and transport, we decided to target two polymers; one with a relatively low molecular weight and a second with a much higher weight. This decision was based on previous work using HBPs with catalytic porphyrin cores [21]. In this work, it was shown that the high-molecular-weight systems did not perform well due to severe steric hindrance around the catalytic groups (caused by the polymers dense globular backbone). However, HBPs with molecular weights around 7500 g/mol provided an optimized microenvironment for substrate binding and catalysis. Furthermore, this lower-molecular-weight HBP possessed an internal micro-environment distinct from the external bulk environment (with respect to its physical and electronic properties). A representative example of the low molecular weight structure targeted for our work is shown in Figure 3. This polymer would ideally have a molecular weight between 7 × 10^3^ and 8 × 10^3^ g/mol. In addition, a larger HBP with a molecular weight above 15 × 10^3^ g/mol would also be synthesized and tested. This larger polymer should provide a much more crowded internal structure, which should present an obvious steric barrier to anion binding and transport.

**Scheme 3:**
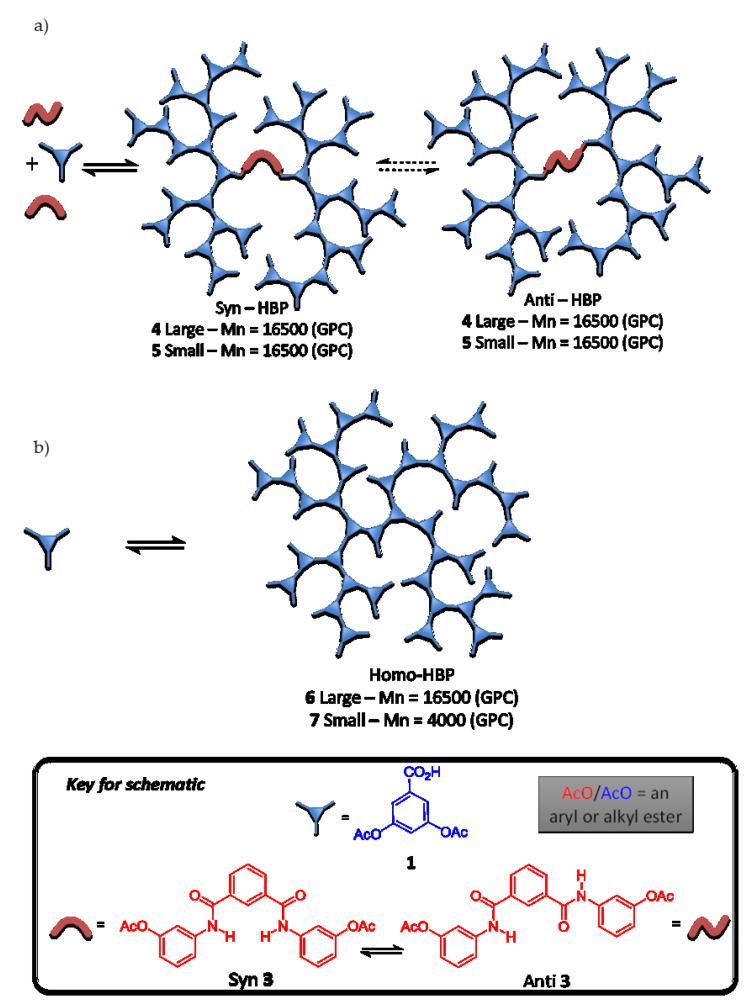
Schematic showing the synthesis of all polymers required for the transport and control studies. (**a**) Synthesis of core functionalized HBPs **4** and **5**. Core functionalized polymers shown in their dynamic syn and anti-conformations. (**b**) Polymerization of monomer **1** to give the homopolymers **6** and **7** required for control experiments.

The synthesis of the target HBPs, including formation of the syn and anti-forms, is shown schematically in Scheme 3. Although not directly correlated, the molecular weight and dispersity can be controlled by varying the isophthalamide core **3** to monomer **1** ratio. That is, if we use a high core-to-monomer ratio, we can predict a relatively low-molecular-weight HBP. Conversely, if we use a lower core-to-monomer ratio, then a higher-molecular-weight polymer can be obtained. For example, if we start with a core to monomer ratio of 1:50, we might predict a degree of polymerization (DP) equal to 50 and a molecular weight around 9 × 10^3^ g/mol. However, theoretical DP and molecular weights are rarely achieved, and polymers with higher DP and molecular weights are almost always obtained [7,8]. Consequently, to target the HBP with the higher molecular weight, we reacted 1 equivalent of the isophthalamide core **3** with 50 equivalents of (3,5-diacetoxy benzoic acid) **1**. Initially, the reaction was simply heated (to establish the reversible transesterification reaction), before placing the reaction under vacuum to remove the acetic acid by-product as it was generated. Removal of the by-product suppresses the back reaction and ensures the reaction is driven towards the HBP product. The crude product obtained was readily dissolved in refluxing THF and purified by precipitating it into ice-cold methanol. The purified product was obtained as a white solid in good yield and as a mixture of the anti and syn conformers **4**, as shown in Scheme 2. Although it was not possible to determine the relative ratio of the syn and anti-forms of the core unit within the HBP, the total level of core incorporation could be calculated from ^1^H NMR using the integration ratio of core to aromatic repeat unit [11,12]. Even though many of the signals from the core unit were coincidental with those from the HBP’s backbone, a broad multiplet at 6.72–6.90 ppm, corresponding to the most shielded –CNH core protons, was clearly visible. Assuming that all HBPs have a single core unit allows us to estimate the level of core incorporation and therefore the DP and molecular weight of the HBP through integration [11]. That is, using an integral of **2** for the –CNH protons and comparing it to the integral from the remaining aromatic protons (allowing for the remaining core protons) permits us to estimate the number of repeat units using the three aromatic protons from each repeat unit. This calculation gave us a core-to-monomer ratio and DP of 1:120. This equates to a molecular weight of 22 × 10^3^ g/mol (using the repeat units’ average molecular weight of 178, which can be derived by assuming a 50% degree of branching).

Analysis using gel permeation chromatography (GPC) gave a number average molecular weight of 16 × 10^3^ (Mn). However, it is well-known that GPC underestimates the molecular weights of dendritic polymers, as the technique is calibrated against linear polystyrene [22]. As such, this result supports the molecular weight obtained by NMR. Furthermore, as the NMR calculation assumes that all HBPs possess a core unit, then the GPC data provides good evidence for very high levels of core incorporation. Our next target was the smaller HBP, with a molecular weight close to the previously reported dense packed limit of 7–8 × 10^3^ g/mol [8]. To achieve this, a core to monomer ratio of 1:20 was used. The product was obtained as white solid in good yield and as a mixture of the anti and syn conformers **5**, as shown in Scheme 3. After purification, the level of core incorporation was estimated using NMR as previously described for the larger HBP. This calculation indicated that a HBP with a DP of around 40 and a molecular weight of 8 × 10^3^ had been synthesized. As before, GPC underestimated molecular weight and generated a Mn value of 6 × 10^3^ g/mol for the smaller HBP **5**. Again, when taking into account the tendency for GPC to underestimate molecular weights of HBPs, we can conclude that the molecular weight obtained by NMR was accurate and the level of core incorporation very high [22].

Two homo HBPs (HBPs without core unit **3**) were also synthesized for use as control systems in the transport studies described below. The use of homo HBPs will allow us to quantify any passive transport from the simple encapsulation within the polymer’s structure and therefore to fully assess the core binding properties of the anion-binding systems **4** and **5**. The two homo polymers **6** and **7** were synthesized and purified as described above, but with the exclusion of an isophthalamide core **3** during the synthetic preparation. Although this approach is simple in principle, it is very difficult to control the molecular weight of the homo polymers without a core unit. Therefore, to obtain the target HBPs, we simply used a crude system of trial and error with respect to polymerization time. Even so, this approach was not reproducible as it was difficult to control the vacuum, which is important with respect to the reversibility of the process. Nevertheless, this method eventually provided us with the control homo HBPs **6** and **7** with GPC indicated molecular weights of 16,500 and 6000, respectively, as shown in Scheme 2. In this respect, they have the same number average molecular weights as the core functionalized systems (by GPC) and can therefore be considered equivalent to the cored HBPs **4** and **5**, with Mn values of 22 × 10^3^ and 8 × 10^3^ by NMR. As such, we now had all of the core functionalized HBPs and control molecules required to fully assess and quantify the transport properties. These are summarized and shown below in Table 1.

### Anion Transport Studies

**Figure 4:**
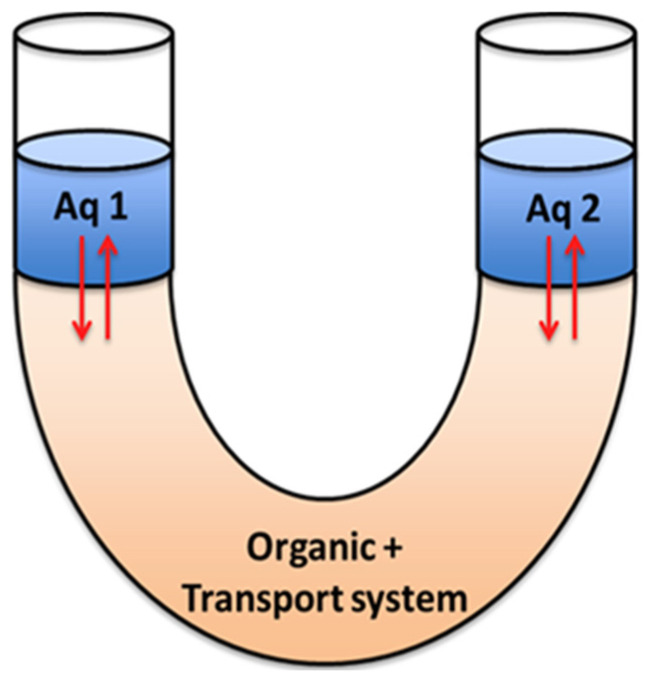
Schematic representation of the Pressman-type model used to measure transport rates.

Anion transport was measured using a conventional U-tube (Pressman) experiment as shown in Figure 4 [14]. In this experiment, the HBP was the carrier and is in the organic phase. The carrier can then transport guests between the two aqueous phases (Aq 1 and Aq 2—Figure 4); in this case, the guest is in the phenolate anion. The process of transport involves the carrier picking up a guest species at the first aqueous interface (Aq 1) and transporting it to the second aqueous phase (Aq 2). The process is reversible and continues until an equilibrium point is reached. Measuring concentration changes over time allows us to determine rates of transport and therefore a relative binding affinity. The deprotonated form of *p*-nitrophenol (*p*-nitrophenolate) is highly water-soluble, poorly soluble in organic solvents, and bright yellow. The intense color allows us to easily determine concentration of the anion using simple UV spectroscopic techniques. *p*-nitrophenol has a dissociation constant (pKa) of 7.16 at 22 °C. To ensure there was enough anion present, we worked at pH 8.00 and used a similar U tube method to the one reported for the determination of sulfate extraction and transport [23]. Initially, control experiments were carried out to determine the extent of simple passive transport (no carrier) and also the transport ability of the two separate components that make up the polymeric transport systems. Specifically, core unit **3** and the homo HBPs **6** and **7**. The amount of simple passive diffusion (i.e., no carrier presents in the DCM layer) was determined by loading a 1 × 10^−4^ M solution of *p*-nitrophenol at pH 8.0, into Aq 1. This solution was a deep and intense yellow color (indicating deprotonation and formation of the anion). Transport was subsequently measured by looking for the same yellow phenolate in Aq 2 and determining concentration at specific time intervals. Concentrations were calculated using the phenolate’s extinction coefficient (22,900 M^−1^ cm^−1^) determined from a simple Beer–Lambert experiment. Using this approach, it was obvious that passive transport of the anion into Aq 2 was extremely poor. Nevertheless, the concentrations obtained at each time interval were subtracted from all future experiments.

**Figure 5:**
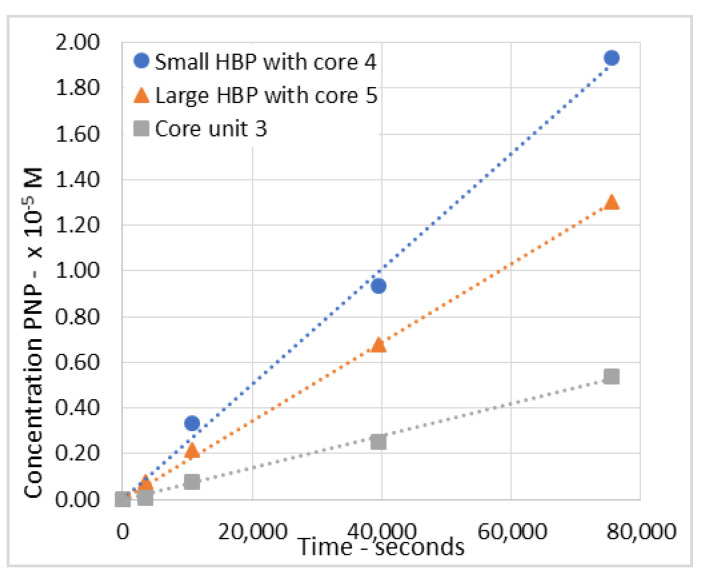
A graph to show UV/Vis data for anion (*p*-nitrophenolate) transport using three different carriers; the small HBP with core **4**, the large cored HBP **5**, and the free receptor **3** (the control).

The experiment was then repeated using HBPs without binding core units (HBPs **6** and **7**), and a similar result was obtained. That is, very little anion (above that observed for passive diffusion) appeared to be transported. The final control experiment involved the core unit **3**. This time, the results showed clear evidence for transport, as the yellow color in Aq 2 became more intense over time. The concentration of phenolate in Aq 2 was plotted with respect to time and the gradient used to quantify transport, which took place with an initial rate of 0.8 × 10^−10^ M s^−1^ (Figure 5). Having carried out all control experiments, we could conclude that passive diffusion and transport using the control HBPs **6** and **7** was negligible. However, the core unit alone was able to transport the phenolate anion reasonably well. The same experimental methods were then used to determine transport efficiency and rates for the small and large HBPs functionalized with anion-binding cores—HBPs **4** and **5**. As with the control core unit **3**, there was clear visual evidence for anion transport, as the color in Aq 2 changed from clear to an intense yellow over time. For both cored HBPs **4** and **5**, plots of phenolate concentration vs. time generated straight lines (over the time period studied) are shown in Figure 5. Initial rates of transport were determined (from the gradient) as 2.6 × 10^−10^ M s^−1^ and 1.7 x10^−10^ M s^−1^ for the small **4** and large **5** HBPs, respectively. The data for all transport molecules and their relative rates are shown in Table 2.

Analysis of the data indicates that the larger HBP **4** has a relatively weak binding affinity, which manifests itself into a slow rate of transport. This larger polymer has a molecular weight and size and therefore possess a compact and crowded interior, which limits the space around the central anion-binding core. The smaller HBP **5** has a molecular weight and size that generates a significantly better binding environment, balanced with respect to rigidity, flexibility, and sterics. As such, the interior has an ordered structure, with a well-defined space around the core unit, and this is the reason it binds the anion with a stronger affinity and a faster rate of transport. Even though our control experiments did not show any significant anion transport for the HBPs without binding cores (HBPs **6** and **7**), we believe the aromatic structure of the polymer’s backbone probably provides some additional electronic contribution to binding. Although binding will be dominated by the H-bonding between the anion and binding core, this will be enhanced by the π−π interactions possible between the polymer and the anion. Finally, when the experiment was repeated at pH 9, the same trend in transport rates was observed. However, as the concentration of phenolate is higher at pH 9, the observed rates were slightly faster.

## 3. Materials and Methods

### 3.1. Reagents and Solvents

All reagents and solvents were obtained from Sigma-Aldrich (UK) and were used as supplied. Dry solvents were obtained from a Grubbs anhydrous solvent dispensing system. All glassware was cleaned and oven-dried overnight (100 °C).

### 3.2. Ultraviolet Spectroscopy

UV absorbance were recorded on an Analytic Jena AG Specord s600 uv/vis spectrometer (Germany) and analyzed using its attached Software (WinASPECT version 1).

### 3.3. Infra-Red Spectroscopy

IR spectra were recorded using a Perkin-Elmer UATR Infrared spectrometer (UK). Spectra were analyzed with Spectrum100 software, and positions of peaks are stated as wave numbers (cm^−1^).

### 3.4. NMR Spectroscopy

All NMR samples were prepared using deuterated solvents supplied by Sigma Aldrich. ^1^H NMR and ^13^C NMR spectra were recorded using a Brucker AV1400 MHz machine (UK). Chemical shifts are quoted using ppm, and coupling constants are quoted in Hertz referenced to residual solvent signals. The NMR spectra were analyzed using Topspin 3.0 NMR software.

### 3.5. Gel Permeation Chromatography 

Analytical THF GPC data was obtained at room temperature using a low molecular weight column (2 × 600 mm PL gel 5 um (500 Å)). Calibration was achieved by using polystyrene standards (Mn 220–1,000,000 Da), and molecular weights are thus reported relative to these specific standards used. The samples were run using Fisher GPC grade THF as a solvent stabilized with 0.025% BHT (which was supplied to the columns by a Waters 515 HPLC Pump at 1.00 mL min^−1^ - UK). Toluene was added to prepared sample as a flow marker, before injection through a 200 µL sample loop with a Gilson 234 Auto Injector (UK). The concentration of a sample was studied using an Erma ERC-7512 refractive index detector. Samples were filtered using Whatman^®^ GD/X syringe filters with a pore size of 0.45 µm prior to analysis.

### 3.6. Synthesis of 3,5-Diacetoxybenzoic Acid (**1**)

A 500 mL round bottom flask was charged with 3,5-dihydroxybenzoic acid (77.00 g, 0.50 mol) and acetic anhydride (200 mL). The reaction mixture was heated to reflux, as the temperature increased the dihydroxy acid gradually dissolved into solution and the mixture was left to reflux for 6 h. A brown solution was obtained containing a small amount of insoluble material; the excess acetic anhydride and acetic acid by-product were removed under reduced pressure, the compound dissolved in refluxing chloroform (200 mL) and filtered hot. Petroleum ether (70 mL) was then added to the mother liquor, precipitating a white solid. The mixture was left overnight, and a white product was isolated by filtration, which was thoroughly washed with petroleum ether. Yield: 40.60 g, 34%; ^1^H NMR (CDCl_3_) δ 10.19 (br s, 1H, -COOH), 7.76 (d, 2H, Ar *o*-CH), 7.22 (t, 1H, Ar *p*-CH), 2.31 (s, 6H, -CH_3_); ^13^C NMR (CDCl_3_, 300 MHz) δ 170.0 (COOH), 168.8 (C=O), 151.3 (*m*-Ar), 131.4 (*i*-Ar), 122.1 (*p*-Ar), 121.0 (*o*-Ar), 21.0 (CH_3_); IR (cm^−1^) 3400–2400, 1765 (COOR), 1687 (COOH), 1603; ES-MS, 237 (M^+^); MP 160–162 °C.

### 3.7. Synthesis of N,N’-Bis-(3-hydroxyphenyl)-isophthalamide (**2**)

3-aminophenol (3.22 g, 0.03 mol) and anhydrous *N,N’*-dimethylacetamide (30 mL) were placed into two-necked round bottom flask equipped with a nitrogen inlet. After the solution was cooled to 0 °C, isophthaloyl chloride (3.00 g, 0.02 mol) was added. The reaction mixture was then stirred for 6 h, after which the temperature was allowed to rise to room temperature. The mixture was poured into 1 L of ice-cold distilled water and filtered under reduced pressure. The crude product was dissolved in the minimum amount of hot ethanol and filtered whilst hot. Then, it was precipitated in ice-cold distilled water and filtered. Finally, the product was placed in a vacuum at 100 °C to ensure complete dryness is achieved. Yield: 4.50 g, 88% ^1^H NMR (CD_3_OD, 400 MHz) δ_H_ 10.30 (s, 2H, OH), 9.49(s, 2H, NH), 8.48 (s, 1H, *Ar-*OCCCHCCO), 8.16 (d, 2H, *Ar*-COCCH), 7.71 (t, 1H, *Ar-*COCCHCH), 7.39 (s, 2H, *Ar-*NHCCHCOH,) 7.20–7.12 (bm, 4H, *Ar-*NHCCHCH), 6.53 (d, 2H, *Ar-*NHCCHCOHCH) ^13^C NMR (CD_3_OD, 400 MHz) δ_c_ 107.8, 111.3, 110.8, 127.5, 129.0, 129.8, 131.0, 135.5, 140.4, 157.7, 165.3 IR (cm^−1^) 3359 (N-H stretch), 3191 (OH stretch), 1647, 1607 (amide C=O stretch), 1541, 1489, 1449, 1393, 684 Elemental analysis%: Carbon (Expected value: 68.96%) Found: 68.53%, Hydrogen (Expected: 4.63%) Found: 4.69% Nitrogen (Expected: 8.04%) Found: 7.94% ES-MS 349 (MH^+^) m.p. 265–266 °C.

### 3.8. Synthesis of N,N’-Bis-(3-acetoxyphenyl)-isophthalamide (**3**)

*N,N’-bis*-(3-hydroxyphenyl) isophthalamide (2.0 g, 5.74 mmol) was dispensed into an oven dried round bottom flask and dissolved in anhydrous pyridine (150 mL). The flask was placed under a nitrogen atmosphere. Acetyl chloride (2.22 mL, 28.28 mmol) was then added. The reaction mixture was then stirred at room temperature for a period of 4 h. Pyridine was removed via Vacuum distillation, yielding brown oil. The resulting brown oil was dissolved in dichloromethane (50 mL) and washed with saturated sodium hydrogen carbonate solution (3 × 10 mL). Trace amounts of acid were removed by washing the organic layer with further portions of water (3 × 10 mL). The organic layer was then reduced in vacuo, and the cream solid was collected and purified by recrystallization. The crude cream solid was dissolved in the minimum amount of hot dichloromethane, and petroleum ether was added drop wise until the yellow solution remained cloudy. The solvent was filtered, yielding the product as cream colored crystals. Yield: 2.01 g, 81% ^1^H NMR (CDCl_3,_ 400 MHz) δ_H_ 9.46 (s, 2H, NH), 8.54 (s, 1H, *Ar-*OCCCHCCO), 8.20 (d, 2H, *Ar*-COCCH), 7.80 (t, 1H, *Ar-*COCCHCHCHCCO), 7.70–7.61 (bm, 4H, *Ar-*CONHCCHCHCHCO, *Ar -*CONHCCHCO), 7.40–7.38 (bm, 2H, *Ar-*CONHCCHCHCHCO), 6.92 (d, 2H, *Ar-*CONHCCHCHCHCO) 2.30 (s, 6H, OCH_3_). ^13^C NMR (CDCl_3,_ 400 MHz) δ_c_ 168.8, 164.9, 151.3, 140.3, 135.4, 130.6, 129.3, 128.8, 126.5, 117.2, 117.2, 117.0, 113.6, 20.1 FTIR (cm^−1^) 3050–3100 (aromatic C-H stretch), 1764 (acetate C=O stretch), 1646, 1603 (amide C=O), 1543, 1483, 1433, 1196, 681 EA%: Carbon (Expected: 66.66%) Found: 65.89% Hydrogen (Expected: 4.66%) Found: 4.43% Nitrogen (Expected: 6.48%) Found: 6.41% ES-MS 433 (MH^+^) m.p. 178–180 °C.

### 3.9. General Method for the Polymerization of 3,5-Diacetoxybenzoic Acid

Various amounts of 3,5-diacetoxy benzoic acid, diphenyl ether, and isophthalamide **3** (when required) were placed into a 250 mL round-bottom flask, which was placed under an inert nitrogen atmosphere. The reaction mixture was then heated to 225 °C and stirred for a period of 45 min. The reaction temperature was then cooled to 180 °C, and a vacuum was applied to the system to remove the acetic acid by-product. The flask was allowed to cool down to room temperature. The brittle foamy polymer obtained was then dissolved in refluxing THF, before precipitating (whilst hot) into ice-cold methanol. The solution was then placed in a freezer overnight. The solid product obtained was collected by filtration and washed with ice-cold methanol. The precipitation procedure was repeated a further two times to give the pure polymer.

### 3.10. Synthesis of “Large” Isophthalamide Cored Hyperbranched Polymer (**4**)

The general method described above was used with the following; 3,5–Diacetoxy benzoic acid (1.0 g, 4.19 mmol), [*N,N’*-*bis*-(3-acetoxyphenyl)-isophthalamide] (0.03 g, 0.08 mmol) and diphenyl ether (1.04 g, 6.09 mmol). Yield: 0.7 g, 70.2%.^1^H NMR (CDCl_3_, 400 MHz) δ_H_ 8.20 (2H, [Core] *Ar*-COCCH), 8.10–7.75 (bm, 1H [Core] *Ar-*COCCHCHCHCCO, 2H [Polymer] *Ar p-*CH), 7.70–7.50 (bm, 4H, [Core] *Ar-*CONHCCHCHCHCO, *Ar -*CONHCCHCO2H), (1H [polymer] Ar-CH dendritic), 7.40–7.18 (bm, 2H, [core] *Ar-*CONHCCHCHCHCO), 2H, [linear polymer] *Ar o*-CH), 6.71–6.95 (bm, 2H, [Core] *Ar-*CONHCCHCHCHCO), 2.25 (bs, 3H, [Polymer] CH_3_). ^13^C NMR (CDCl_3_, 400 MHz) δ_C_ 168.8, 162.8, 151.2, 130.9, 130.7, 129.7, 123.2, 121.3, 120.9, 118.9, 21.0 FTIR (cm^−1^) 3096, 2163, 2031 (aromatic C-H stretch), 1743 (ester C=O stretch), 1441, 1268, 749 GPC M_n_ = 16 × 10^3^ g·mol^−1^, M_w_ = 41 × 10^3^ g·mol^−1^.

### 3.11. Synthesis of “Small” Isophthalamide-Cored Hyperbranched Polymer (**5**)

The general method described above was used with the following; 3,5–Diacetoxy benzoic acid (2.0 g, 8.39 mmol), [*N,N’-bis*-(3-acetoxyphenyl) isophthalamide] (0.01 g, 0.21 mmol) and diphenyl ether (2.1 g, 12.33 mmol). Yield: 1.68 g, 84%. ^1^H NMR (CDCl_3_, 400 MHz) δH 8.18 (2H, [Core] Ar-COCCH), 8.10–7.75 (bm, 2H [Polymer] Ar *p-*CH, 1H [Core] Ar-COCCHCHCHCCO,), 7.65–7.50 (bm, 2H [core] Ar -CONHCCHCO), (1H [polymer] Ar-CH dendritic), 7.40–7.18 (bm, 2H, [core] Ar-CONHCCHCHCHCO), 2H, [linear polymer] Ar o-CH), 7.30–7.20 (bm, 2H, [Core] Ar-CONHCCHCHCHCO), 2.25 (bs, 3H, [Polymer] CH3). ^13^C NMR (CDCl3, 400 MHz) δC 168.8, 162.8, 162.7, 151.2, 139.1, 130.9, 130.6, 129.3, 120.9, 21.0 FTIR (cm-1) 3114–3064 (aromatic C-H), 1772 (ester C=O stretch) 1374, 1277, 772. GPC M_n_ = 6 × 10^3^ g·mol^−1^, M_w_ = 8.5 × 10^3^ g·mol^−1^.

### 3.12. Synthesis of the “Large” and “Small” Control Hyperbranched Polymers (**6** and **7**)

The general method described above was used with the following; 3,5–Diacetoxy benzoic acid (2.38 g, 0.02 mol) and diphenyl ether (3.00 g, 0.02 mol). After the initial heating at 225 °C, the reactions were heated at 180 °C (under vacuum) for 4 and 6 h, for the “small” and “large” HBPs, respectively. Yield: 68–70%. ^1^H NMR (CDCl_3_, 400 MHz) δ_H_ 8.18–7.96 (br d, 1H, [Polymer] Ar *p-*CH), 7.95–7.80 (br d, 2H, [Polymer] Ar *o-*CH), 7.52(s, Ar-CH dendritic), 7.41 (s, Ar-CH linear), 7.25 (s, Ar-CH terminal), 2.38 (br s, 3H [Polymer] CH_3_). ^13^C NMR (CDCl_3_, 400 MHz) δ_C_ 168.8, 162.8, 151.2, 130.1, 130.8, 121.3, 120.9, 21.0 FTIR (cm^−1^) 2970, 2039 (aromatic C-H stretch), 1770 (ester C=O stretch) 1279,1185; GPC for the “large” HBP **6**, M_n_ = 16,500 M_w_ = 33,000. GPC for the “small” HBP **7**, M_n_ = 6 × 10^3^ M_w_ = 13.2 × 10^3^.

### 3.13. Anion Transport Studies 

The method used was adapted from a literature method [23]. A 15 mm diameter U tube was prepared as follows: 100 mL of a dichloromethane solution containing 1 × 10^−4^ M of the transport agent or control (for HBPs, concentrations were calculated using Mn values from NMR, which in turn were based on, or estimated from, the level of core incorporation.) A small stirrer bar was then placed into the U tube. On the left-hand side of the U tube, 100 mL of a 1 × 10^−4^ M solution of *p*-nitrophenol in buffer (pH 8.00, 0.01 M phosphate) was carefully added by pipette. On the right-hand side, 100 mL of buffer (pH 8.0, 0.01 M phosphate) was carefully added. Gentle stirring was then applied and the right-hand side periodically analyzed by UV to estimate the concentration of phenolate anion (all UV samples were returned to the right hand side after analysis). 

## 4. Conclusions

The results have described an efficient method for synthesizing two core functionalized hyperbranched polymers. Specifically, each contained an anion-binding core within the interior of a globular dendritic structure. These dendritic polymers, which only differed in their molecular weight and size, were able to bind and transport a water-soluble anion across an organic phase and between two aqueous phases. Control experiments demonstrated that passive diffusion was negligible, as was transport using non-cored homo HBPs. In addition, a control experiment using the anion-binding core without the HBP showed some ability to transport the anion, and this was used as a baseline to assess the potential of the two core functionalized HBPs. Binding and transport experiments using the two HBPs gave transport rates that outperformed those of the simple anion-binding receptor. In the worst case, the HBPs transported the anion with a rate 200% faster than the simple anion-binding receptor (the control reaction). As such, it is clear that the microenvironment provided by the dendritic polymer provides the optimum steric and electronic surroundings for the anion investigated. Although both HBPs performed well, it was the smaller of the two polymers that performed best, transporting the anion with a rate that was 50% greater than the larger HBP (and 320% faster than the simple anion-binding core). Whilst both dendritic polymers could protect the anion from the external organic phase, by encapsulating and binding it at the center of the HBP, the monomer units within the larger HBP were significantly more densely packed, which created a steric barrier that limited the anion’s access to the central binding core. Although control experiments concluded that the homo HBPs (HBPs without binding cores) could not facilitate significant anion transport, we suggest that the aromatic monomers can in fact contribute some of the binding energy. Overall, the methodology demonstrates clear potential and future capacity for HBPs to act as encapsulation vehicles (capable of binding and transporting a variety of guests).

## Figures and Tables

**Table 1:** Molecular weight data for anion transport and control experiments.

	Molecule	Mn-NMR	Mn-GPC
**HBP anion transport molecules**	Large HBP with core 4	22 × 10^3^	16.5 × 10^3^
	Small HBP with core 5	8 × 10^3^	6 × 10^3^
**Control molecules**	Core unit 3	/	/
	Large HBP no core 6	22 × 10^3^ *	16.5 × 10^3^
	Small HBP no core 7	8 × 10^3^ *	6 × 10^3^

* Molecular weights shown could not be calculated by NMR (as there is no core for reference) but are an estimate based on the Mn values from GPC for the core functionalized molecules (**6** and **7**).

**Table 2:** Rates and relative rates of anion transport using the anion-binding core containing molecules **3**, **4**, and **5**.

	Rate of Transport (10^−10^ M s^−1^)	Relative Rate
Core unit **3** (the control)	0.8	1.0
Small HBP with core **5**	2.6	3.2
Large HBP with core **4**	1.7	2.1

## Data Availability

Not applicable.

## Sample Availability

Samples of the compounds are no longer available from the authors.
